# Predictive modeling with machine learning for low birth weight in the state of São Paulo: a retrospective population-based study

**DOI:** 10.1590/1516-3180.2025.3373.04052026

**Published:** 2026-07-24

**Authors:** Allbert Velleniche de Aquino Almeida, Adriana de Oliveira Ribeiro dos Santos, Luiz Fernando Costa Nascimento

**Affiliations:** I Departamento de Engenharia, Faculdade de Engenharia e Ciências, Universidade Estadual Paulista “Júlio de Mesquita Filho” (Unesp), Guaratinguetá (SP), Brazil.; II Departamento de Medicina, Universidade de Taubaté, Taubaté (SP), Brazil.; III Departamento de Engenharia, Faculdade de Engenharia e Ciências, Universidade Estadual Paulista “Júlio de Mesquita Filho” (Unesp), Guaratinguetá (SP), Brazil.

**Keywords:** Infant, low birth weight, Machine learning, Predictive learning model, Logistic models, Random forest, Infant, newborn, Pregnancy, Prenatal care, Health policy, Data mining, Artificial intelligence

## Abstract

**BACKGROUND:**

Low birth weight (LBW) is a major public health concern associated with increased neonatal morbidity and mortality.

**OBJECTIVE:**

This study was aimed to develop a predictive learning model using machine learning techniques to identify LBW from live birth data.

**METHODS:**

A retrospective population-based study was conducted using data from the Brazilian Live Birth Information System (SINASC), which includes all live births registered in the state of São Paulo between 2019 and 2023. After data curation and exclusion of records with missing or unknown information, a total of 2,548,570 live births were analyzed. Predictors included maternal sociodemographic characteristics, obstetric history, prenatal care indicators, and newborn characteristics. Logistic regression and random forest models were trained using an 80/20 train–test split, with class imbalance addressed through class weighting. Model performance was evaluated using accuracy, precision, recall, F1-score, and area under the receiver operating characteristic curve (AUC-ROC).

**RESULTS:**

The analysis showed that the balanced logistic regression model performed best in terms of sensitivity (0.688), F1-score (0.567), and AUC-ROC (0.850). A proportional analysis of the variables between the LBW and normal-weight groups revealed a higher prevalence of LBW among mothers with low education levels, without a partner, of Black or Brown ethnicity, aged ≤ 19 years or ≥ 35 years, with multiple pregnancies, with a reduced number of prenatal consultations, and with late initiation of prenatal care.

**CONCLUSIONS:**

The findings highlight the importance of large-scale data analysis supported by machine learning techniques to inform public policies aimed at preventing LBW and promoting maternal and child health.

## INTRODUCTION

Low birth weight (LBW), defined by the World Health Organization (WHO) as birth weight < 2,500 g, is widely recognized as a key indicator of neonatal morbidity and mortality and is associated with short- and long-term complications in child development.^1^ Approximately 15% of births worldwide^2^ and approximately 10% in Brazil^3^ are LBW, underscoring the magnitude of the problem and the importance of preventive strategies.

Several factors are associated with LBW, including unfavorable socioeconomic conditions, extreme maternal age, low educational level, obstetric history, prematurity, multiple pregnancies, and inadequate prenatal care.^4,5,6,7^ Recent studies on prematurity and cesarean delivery by Abdi et al. and Rezaei et al.^8,9^ highlight advanced maternal age, obstetric history, and maternal comorbidities as relevant risk factors.^4,5,6,7^

Many of these factors can be effectively addressed during pregnancy if identified early, reinforcing the importance of vigilant and qualified prenatal monitoring.^5,10,11^

In recent years, the application of artificial intelligence (AI) techniques, especially machine learning (ML), has emerged as an innovative and effective approach in public health, enabling the analysis of large volumes of data and the development of robust predictive models.^8,9,12,13,14,15^ Models based on algorithms such as decision trees, logistic regression, artificial neural networks, and random forest have been successfully used to predict clinical outcomes, including LBW.^8,9,16,17,18^

Recent studies have shown that predictive models using population data can achieve good accuracy in the early identification of pregnancies at risk for LBW, aiding in the planning of targeted interventions and efficient resource allocation.^19,20^ In Brazil, the Live Birth Information System (SINASC) provides a comprehensive, nationwide database on births, which includes relevant information regarding the mother, the pregnancy, the delivery, and the newborn. This makes it a valuable source for epidemiological studies and predictive modeling.^3^

Therefore, this study aimed to develop and validate ML models capable of predicting LBW occurrences in the state of São Paulo using SINASC data.

## METHODOLOGY

This study was conducted using data from the state of São Paulo, which comprises 645 municipalities and has the largest population among Brazilian states, with approximately 45.9 million inhabitants according to estimates by the Instituto Brasileiro de Geografia e Estatística (IBGE).^21^ This territorial and demographic diversity makes the state a representative sample of the national population with varying socioeconomic contexts, levels of healthcare access, and human development indicators. These characteristics justify the choice of São Paulo as the study setting, allowing for broader analyses with the potential to be generalized to other regions of the country.

This applied research followed a methodological procedure consisting of problem definition, literature review, research design, data collection, data analysis, interpretation of results, and presentation of conclusions. Each stage required careful attention and methodological rigor to ensure the quality and relevance of the results.

The methodology adopted a quantitative and qualitative approach, combining the analysis of documentary data obtained from public databases with the use of ML techniques for prediction.

Data from SINASC referring to São Paulo covering five full years of records (from 2019 to 2023) were used. With approximately 600,000 births per year in the state, the sample included a wide range of cases, ensuring representativeness and diversity.

The SINASC data include numerous variables regarding live births, some of which are repeated (e.g., maternal education, which is recorded as three different variables with historical and categorized formats). To optimize processing and avoid redundant information, highly correlated variables were eliminated. The selection of the variables considered in the study was based on those commonly used in previous studies on LBW prediction and in those that were effectively available in SINASC records. This strategic selection contributed to the development of leaner, more interpretable models with good predictive performance.

Many studies have employed variables from SINASC to analyze LBW, including maternal age, marital status, education, race/color, occupation, parity, pregnancy type (single or multiple), delivery type (cesarean or normal), previous fetal loss, number of prenatal visits, month when prenatal care started, gestational age, delivery location, and newborn sex and weight. Other common variables include the Apgar score and the municipality of residence. These sociodemographic, obstetric, and perinatal factors directly influence birth outcomes and have been widely used in LBW predictive models.

This study selected variables related to maternal medical history, socioeconomic factors, and prenatal health indicators to ensure the inclusion of relevant predictors for LBW. Selected variables include:

- **Maternal medical history:** age at delivery, number of previous child deaths, parity (birth order), and pregnancy type (single, twin, triplet, or more).
- **Socioeconomic factors:** education level (categories 0 to 5 according to SINASC), marital status (married/stable union and widowed/single/separated), and maternal race/color (White, Black, Yellow, Brown, Indigenous).
- **Prenatal health indicators:** gestational age category (preterm, term, post-term); number of prenatal visits; month prenatal care began; detected congenital anomalies (yes/no); and newborn sex (male/female).

Prior to model development, a rigorous data curation process was performed to ensure data quality and analytical consistency. All records containing missing values, inconsistent entries, or including categories such as “ignored“ or “not informed“ for key variables were excluded. After this selection process, 2,548,570 valid live birth records from an initial dataset of 2,677,170 records were retained.

Feature selection was guided by a combination of epidemiological relevance and data availability within the SINASC. Variables with high redundancy or strong collinearity—such as multiple representations of maternal education—were evaluated, and only the most informative one was retained. This approach was aimed to reduce dimensionality, improve model interpretability, and avoid overfitting. No automated feature selection algorithms were applied; instead, selection was based on prior literature and domain knowledge regarding established risk factors for LBW.

In the preprocessing step, a “low weight“ binary variable was defined by assigning a 0 to births with normal weight (≥ 2,500 g) and a 1 to those with LBW (< 2,500 g).

SINASC data were accessed via DATASUS, converted into comma-separated values (.csv) files using TabWin version 4.15 (Departamento de Informática do Sistema Único de Saúde – DATASUS, Brasília, DF), and stored in Google Drive to facilitate collaborative analyses use through Google Colab.

Data processing and analysis were performed using Python in Google Colab, which can handle large datasets with virtual machines. Packages such as NumPy and Pandas supported data processing, and scikit-learn was used to implement ML algorithms.

Several ML algorithms were tested, including logistic regression and random forest. Logistic regression is a widely used statistical model for binary classification, whereas random forest combines multiple independent decision trees to generate a final prediction. These models were selected because they are commonly used for classification tasks in epidemiological and health-related studies.

The curated dataset was randomly partitioned into a training set (80%) and an independent holdout test set (20%). The training set was used exclusively for model fitting and internal cross-validation procedures, whereas the test set was reserved for final performance evaluation. In ML models, especially when working with imbalanced datasets, it is important to apply balancing techniques to prevent the majority class from dominating the results. Therefore, balancing techniques were applied to the algorithmic models to ensure that the model learns to identify relevant features from both classes, thereby improving precision and sensitivity, and making it possible to apply the results to other datasets.

ML model evaluation was conducted using metrics widely employed in classification tasks, with emphasis on the confusion matrix, which allows for a detailed analysis of performance in terms of true and false positives and negatives.

Metrics such as accuracy, precision, recall, and the F1-score are obtained from the confusion matrix, which allow the evaluation of the model predictive performance. Accuracy indicates the proportion of correct predictions, precision evaluates the quality of positive predictions, and sensitivity measures the ability to correctly identify true LBW cases. Finally, the F1-score balances precision and sensitivity, and is particularly useful when class imbalance is present.

Additionally, the complementary metric area under the receiver operating characteristic curve (AUC-ROC) was used to evaluate model performance. Values closer to 1 indicate excellent discriminatory ability, whereas those near 0.5 indicate a prediction ability equivalent to random chance.

All reported performance metrics, including accuracy, precision, recall, F1-score, and AUC-ROC, were calculated exclusively using the holdout test set. Receiver operating characteristic (ROC) curves were generated using predicted probabilities obtained from the test data, ensuring unbiased assessment of the discriminative performance of the model. These methodological approaches were aimed to ensure a comprehensive evaluation of the performance that considers not only the overall accuracy but also the effectiveness in correctly identifying LBW.

Finally, an exploratory data analysis was conducted to investigate the distribution of the variables used in the study. This step was aimed to evaluate the behavior of maternal and neonatal characteristics present in the dataset, allowing for the identification of possible associations with LBW occurrence. To this end, graphical representations accompanied by proportional analyses between comparison groups were used to address specific questions related to the phenomenon studied.

### Research ethics committee approval

This study was approved by the Research Ethics Committee of Universidade de Taubaté on February 17, 2011, under protocol number 009/11.

## RESULTS

Of the 2,548,570 valid records of live births included in the analysis, 2,307,505 (90.54%) corresponded to newborns with normal weight and 241,065 (9.46%) to cases of LBW (< 2,500 g).

The training set consisted of 2,038,856 records, whereas the remaining 509,714 records were used for testing/validation.

**Figure 1:**
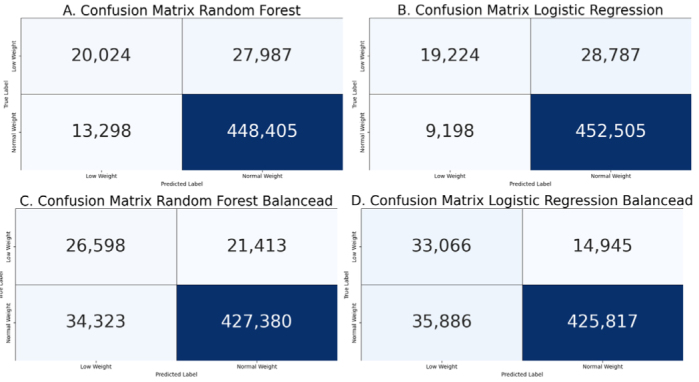
Confusion matrices for the random forest and logistic regression models applied to the prediction of low birth weight (LBW) in the State of São Paulo from 2019 to 2023.

After model training, they were applied to the test/validation dataset. The following results were obtained for the random forest and logistic regression models: **Figure 1 (A and B**) presents the confusion matrices for each model, showing their ability to achieve the highest accuracy in predicting all cases. Training was then performed with balanced classes using the command *class_weight = ‘balanced‘*, in which the algorithm automatically adjusts class weights based on the inverse frequency of each class in the dataset. The results show a balance between the classes, enabling the prediction of specific LBW cases for each model used (**Figure 1, C and D**).

After training, the performance of the random forest and logistic regression models was evaluated using widely accepted metrics in the ML literature, including accuracy, precision, recall, and the F1-score, both before and after balancing classes (**Table 1**). Although both models showed higher accuracy without balancing, they did not demonstrate good recall performance under this condition.

**Table 1:** Evaluation metrics for the random forest and logistic regression models before and after balancing

Model	Accuracy	Precision	Recall	F1-score
Random forest	0.919	0.601	0.417	0.492
Random forest (balanced)	0.89	0.436	0.554	0.488
Logistic regression	0.925	0.678	0.399	0.502
Logistic regression (balanced)	0.901	0.483	0.688	0.567

**Figure 2:**
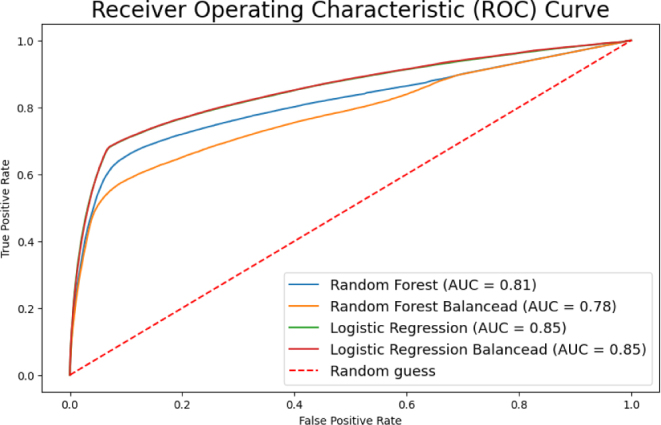
Areas under the receiver operating characteristic curve (AUC-ROC) for the random forest and logistic regression models under different balancing scenarios used to predict low birth weight occurrence in the State of São Paulo between 2019 and 2023.

All evaluated models showed AUC ROC values considered relevant to the objectives of the study, both with and without balancing. Among them, the logistic regression model achieved the best performance, outperforming the random forest model in the evaluated metrics (**Figure 2**).

**Table 2** presents the distribution of variables corresponding to maternal medical history, socioeconomic factors, and prenatal health indicators in the LBW and normal-weight groups.

**Table 2:** Distribution of low birth weight and normal birth weight cases for each of the variables considered in the model

Variables	< 2,500 g (%)	≥ 2,500 g (%)
Maternal age		
≤ *19 years*	10.457	9.181
*20–34 years*	65.489	70.710
≥ *35 years*	24.054	20.109
Type of pregnancy		
*Singleton*	82.610	99.004
*Twin or more*	17.390	0.996
Parity		
*Multiparous*	59.479	62.463
*Nulliparous*	40.521	37.537
Previous child deaths		
*None*	76.242	80.029
*One or more*	23.758	19.971
Maternal marital status		
*With partner*	53.017	56.515
*Without partner*	46.983	43.485
Maternal race		
*White*	51.960	53.711
*Black*	8.297	7.259
*Asian*	0.536	0.537
*Brown*	39.111	38.380
*Indigenous*	0.097	0.113
Maternal education		
*No education or elementary*	16.600	13.297
*High school*	56.839	58.526
*Higher education*	26.561	28.176
Month of prenatal care start		
*0–3 months*	86.290	88.203
≥ *4 months*	13.710	11.797
Prenatal consultations		
*0–6*	37.835	16.010
*7–12*	57.473	75.803
≥ *12*	4.692	8.186
Newborn sex		
*Female*	53.154	48.429
*Male*	46.846	51.571
Identified anomaly		
*No*	96.571	98.898
*Yes*	3.429	1.102

Initially, the models were trained without dataset balancing, reflecting the distribution of real data, which is typically imbalanced between classes (low weight and normal weight). Under this condition, logistic regression achieved the highest accuracy (92.5%) and highest precision (67.8%), indicating good ability to correctly identify positive cases. However, the sensitivity (recall) was relatively low (39.9%), demonstrating that the model had difficulty identifying all true LBW cases, which is common when imbalanced is present.

The random forest model, in turn, showed a slightly lower accuracy (91.9%), lower precision (60.1%), and a sensitivity of 41.7% without balancing. This indicates that the overall performance was similar to that of the logistic regression model; however, it still had difficulties in the adequate identification of cases from the minority class (LBW).

A significant change was observed in the metrics after balancing, especially regarding sensitivity, which is crucial in public health contexts where detecting positive cases (LBW in this case) is a priority.

The balanced logistic regression model showed a significant increase in recall (68.8%), indicating an enhanced ability to correctly identify LBW cases. Despite decreases in precision (48.3%) and accuracy (90.1%), the balanced model achieved the highest F1-score (56.7%), reflecting a more efficient balance between precision and recall.

The balanced random forest model also showed improvement in recall (55.4%) compared to the original model, although accuracy (89%) and precision (43.5%) were decreased. The F1-score was 48.8%, slightly lower than that of the balanced logistic regression model.

Regarding AUC-ROC logistic regression stood out once again, both with and without balancing, achieving a value (85%) that reflects good discriminative ability. The AUC-ROC values for the random forest model without (81%) and with balancing (78%), in contrast, suggest a slight loss of discriminative capacity after balancing.

The precision–recall curves confirm these findings. The balanced logistic regression model achieved the best performance (60%), followed by the logistic regression model without balancing (57%). The random forest models showed worse performances (54% and 52%, respectively).

These results demonstrate that the balancing technique positively impacts sensitivity, and that the balanced logistic regression model proved to be the most appropriate in the analyzed context by achieving the best balance among the metrics. Moreover, the consistent performance in terms of AUC-ROC confirms the robustness of this model for LBW prediction.

## DISCUSSION

The present study demonstrated the usefulness of machine learning approaches for identifying factors associated with low birth weight. Among the evaluated models, logistic regression showed the most favorable overall performance, particularly after class balancing, suggesting that simpler and more interpretable algorithms may perform as well as or better than more complex methods in population-based epidemiological datasets. The improvement observed after balancing reinforces the importance of addressing class imbalance when predicting relatively infrequent outcomes such as low birth weight.

Several maternal, socioeconomic, and prenatal care factors were associated with low birth weight and were consistent with findings reported in previous studies.

Extreme maternal ages (≤ 19 years and ≥ 35 years) were associated with a higher occurrence of LBW, corroborating the results reported by Vale et al.,^5^ Victor et al.,^20^ Abdi et al.^8^ and Rezaei et al.,^9^ who identified maternal age as one of the main predictors of LBW. Parity, represented by the first pregnancy, was also observed to be associated to a higher proportion of LBW, as described by Vale et al.^5^ Another relevant factor was a history of previous child deaths, indicating that women who had experienced the death of a previous child were more likely to have newborns with low birth weights, as pointed out by Ranjbar et al.^7^ Similarly, multiple pregnancies (twins or triplets) were associated with a higher proportion of LBW, as previously reported by Morais et al.^22^ and in agreement with prematurity-related outcomes described by Abdi et al.^8^ The analysis of socioeconomic factors demonstrated that pregnant women with low levels of education, especially those with no formal schooling or elementary education only, had a higher proportion of LBW, which aligns with the findings of Pollob et al.,^19^ Vale et al.,^5^ and Morais et al.^22^ Additionally, marital status revealed that pregnant women without partners are more likely to have newborns with LBW, in agreement with the results reported by Vale et al.^5^ and Victor et al.^20^ Regarding maternal self-declared race, pregnant women who identified as Black and Brown had a higher proportion of LBW, presumably due to social inequalities that directly impact maternal and child health, as previously highlighted by Vale et al.^5^

Regarding variables related to prenatal care, it was noted that LBW cases were significantly more frequent in pregnant women who had had up to six consultations. This result aligns with those reported by Nascimento et al.,^10^ Victor et al.,^20^ and Suarez and Santana,^11^ and is further supported by Abdi et al.,^8^ who emphasized the role of inadequate prenatal follow-up in adverse neonatal outcomes.. Beginning prenatal care by the third month of pregnancy was associated with a higher proportion of newborns with normal weight. Another relevant finding was that female newborns had a higher prevalence of LBW, which has also been reported by Nascimento et al.,^10^ Vale et al.,^5^ and Suarez and Santana.^11^ Additionally, the presence of anomalies identified during pregnancy was strongly associated with adverse outcomes, as highlighted in studies focused on identifying prenatal risk factors, such as that by Morais et al.^22^

This study has some limitations that should be considered when interpreting the results. As it is based on a secondary data source (SINASC), the possibility of potential typing errors, incomplete or incorrect data entry, underreporting, or inaccurate classification of gestational age or birth weight cannot be ruled out, and this is also the case for errors in the measurement of clinical variables or the omission of relevant information by the professionals responsible for data recording.

Internal validity was strengthened through the use of a large, population-based dataset, standardized data collection procedures inherent to SINASC, and strict separation between the training and test datasets. The application of class balancing techniques and the use of multiple evaluation metrics further contributed to a robust assessment of model performance, particularly in the identification of LBW cases.

External validity is supported by the comprehensive coverage of SINASC, which includes virtually all live births that took place in the state of São Paulo during the study period. Given the demographic, socioeconomic, and healthcare diversity of this state, the findings may be generalizable to other Brazilian regions with similarly structured health systems. However, caution is warranted when extrapolating the results to settings with substantially different levels of healthcare access or data quality, or a different population profile. Future studies should assess model performance using external datasets from other states or countries to further validate its generalizability.

## CONCLUSION

The results showed that the balanced logistic regression model achieved the best overall performance, standing out as a particularly appropriate approach for scenarios with class imbalance, as observed in the present study.

In parallel, the analysis of variable distribution confirmed evidence from the previous literature by identifying factors such as extreme maternal age, low educational attainment, absence of a partner, Black or Brown maternal race/ethnicity, multiple pregnancies, history of previous child deaths, female sex for the newborn, pregnancy anomalies, reduced number of prenatal visits, and late initiation of prenatal care as being associated with a higher proportion of LBW deliveries.

Notwithstanding the limitations of the study, the use of ML techniques proved effective in predicting LBW and analyzing relevant patterns in the data. The present study contributes to strengthening maternal and child health surveillance efforts by providing evidence-based insight that can be used to guide preventive strategies aimed at reducing LBW rates and promoting better health for mothers and newborns.

## Data Availability

The datasets analyzed in the current study are publicly available from the Sistema de Informações sobre Nascidos Vivos database, maintained by the Brazilian Ministry of Health, through the DATASUS.
